# Are genital examinations necessary for STI screening for female sex workers? An audit of decriminalized and regulated sex workers in Melbourne, Australia

**DOI:** 10.1371/journal.pone.0231547

**Published:** 2020-04-16

**Authors:** Evelyn M. Turek, Christopher K. Fairley, Catriona S. Bradshaw, Marcus Y. Chen, Lenka A. Vodstrcil, Anthony Snow, Ria Fortune, Eric P. F. Chow

**Affiliations:** 1 Melbourne Sexual Health Centre, Alfred Health, Melbourne, Victoria, Australia; 2 Central Clinical School, Monash University, Melbourne, Victoria, Australia; 3 Centre for Epidemiology and Biostatistics, Melbourne School of Population and Global Health, University of Melbourne, Melbourne, Australia; University of Pretoria, SOUTH AFRICA

## Abstract

**Background:**

The Victorian legislation prohibits sex workers from working when they have visible anogenital herpes or warts. The aim of this study was to determine the proportion of asymptomatic female sex workers (FSW) diagnosed with anogenital herpes or warts by genital examination.

**Methods:**

We analysed all computerised medical records of consultations with FSW at the Melbourne Sexual Health Centre (MSHC) in 2018. All asymptomatic sex workers were offered screening sexually transmitted infections (STIs) and a genital examination to identify visible anogenital herpes or warts at MSHC. FSW consultations were categorised into either ‘asymptomatic’ or ‘symptomatic’ based on the presence of symptoms reported by the FSW to the triage nurse. The proportion of asymptomatic FSW diagnosed with visible anogenital herpes or warts during a routine screening examination was calculated.

**Results:**

In 2018, 4055 consultations were provided to 1979 FSW. 3406 of these consultations were asymptomatic and all were examined by an experienced clinician for signs of STIs. Of these 3406 asymptomatic consultations, seven FSW (0.21%, 95% CI: 0.08% to 0.42%) were diagnosed with visible anogenital herpes and/or warts following a genital examination. Four were diagnosed with warts (0.12%, 95% CI: 0.03% to 0.30%), two with herpes (0.06%, 95% CI: 0.01% to 0.21%) and one with both herpes and warts (0.03%, 95% CI: 0.001% to 0.16%).

**Conclusion:**

Based on these data, approximately 500 asymptomatic FSW would need to be examined to identify one case of anogenital herpes or warts. Genital examinations consume considerable clinical resources, increase the duration of consultations and provide essentially no significant benefit to the mandated testing for gonorrhoea, chlamydia, HIV and syphilis. Our clinic will use self-collected samples and no longer examine FSW who are asymptomatic.

## Introduction

In Victoria, Australia, sex work is a licenced and regulated industry governed by the Sex Work Act 1994 and the Sex Work Regulations 2016 [1]. Under this legislation, it is an offence for a sex worker to work if they are aware they have a sexually transmitted infection (STI) including HIV. By law, sex workers working in Victoria in Australia are required to have mandatory HIV and STI screening every three months [2]. The Sex Work Regulations (2016) defines STIs as ‘HIV, infectious syphilis, gonorrhoea, chlamydia, chancroid, donovanosis, genital and anal herpes (when lesions visible) or genital and anal warts (when lesions visible)’. Current legislation defines ‘adequate STI screening’ as blood tests and swab tests but does not specifically describe a need for genital examinations.

The Melbourne Sexual Health Centre (MSHC) is the main provider of free STI testing and treatment for sex workers in Victoria, Australia. The MSHC protocol includes a genital examination as a component of screening for female sex workers (FSW) in order to identify visible herpes and warts, including those who self-report no symptoms during triage. However, these examinations take up significant clinic and staff time and resources that could otherwise be allocated to other clients at a greater risk of HIV/STI. Given FSW in Australia have a substantially lower prevalence of HIV/STI than women who are not sex workers attending sexual health clinics [2–4], it is questionable whether it is necessary to perform genital examinations among FSW who are asymptomatic. This study aimed to determine the proportion of asymptomatic FSW diagnosed with anogenital herpes or warts during routine genital examinations.

## Methods

We conducted a retrospective audit of all consultations of FSW attending MSHC between 1^st^ January and 31^st^ December 2018. MSHC is the major public sexual health clinic in Melbourne that provides over 50,000 consultations annually [5]. MSHC operates as a walk-in triage service. During triage, clients are asked specifically about the presence of common anal and genital symptoms such as pain, discharge and ‘lumps or bumps’. Based on the self-reporting of symptoms, clients are categorised as either asymptomatic or symptomatic. Clients are then seen by a doctor or nurse. As per the MSHC protocol, all FSW attending for HIV/STI screening would receive a genital examination regardless of the presence of symptoms.

FSW were defined as cis-gender females who self-identified as a current sex worker and attended MSHC for a sex worker ‘certificate-of-screening’ for work. FSW who reported their gender as ‘transgender’ or ‘other’ were excluded from this analysis. The proportion of asymptomatic FSW diagnosed with anogenital herpes and/or warts was calculated using the clinic’s specific codes for these conditions. Clinicians must choose from a pre-determined list of specific clinical diagnostic codes (including a code for herpes and warts) to be able to complete a consultation. These diagnostic codes have been used in many studies and have proven to be accurate [6]. All coded cases of herpes and warts found amongst asymptomatic FSW during an examination were subject to a medical file review for confirmation. Descriptive statistics and proportions with 95% confidence intervals (CI) were calculated using Stata (Version 14, College Station, TX: StataCorp LP.). This study was approved by the Alfred Hospital Ethics Committee, Melbourne, Australia (number 244/19).

## Results

In 2018, 4055 consultations were provided for 1979 FSW attending MSHC, with a median age of 30 years (interquartile range 25 to 37). Less than half (47%; n = 928) of FSW were born in Australia. Of these 4055 consultations, 3406 (84%) were consultations where FSW were asymptomatic, and 649 (16%) were consultations where FSW were symptomatic. Among these 3406 consultations where FSW were asymptomatic, seven FSW (0.21%, 95% CI: 0.08% to 0.42%) were diagnosed with anogenital herpes and/or warts following a genital examination. This included four FSW diagnosed with warts (0.12%, 95% CI: 0.03% to 0.30%), two FSW diagnosed with herpes (0.06%, 95% CI: 0.01% to 0.21%) and one FSW diagnosed with both herpes and warts (0.03%, 95% CI: 0.001% to 0.16%). Of the 649 consultations where FSW were symptomatic, 18 FSW (2.8%, 95% CI: 1.7% to 4.3%) were diagnosed with anogenital warts and 33 (5.1%, 95% CI: 3.5% to 7.1%) with anogenital herpes.

Chart review was performed on the seven asymptomatic FSW diagnosed with anogenital herpes and/or warts. Of the four FSW diagnosed with warts, one reported she had lesions during the clinical consultation after triage; and the other three were not aware of the presence of their warts. Of the two FSW diagnosed with herpes, one had a past history of genital herpes; and the other was thought on the day to have anal dermatitis by the clinician but laboratory testing for herpes was positive. The one FSW diagnosed with both herpes and warts reported no symptoms during triage but did report symptoms to a clinician when prompted during the examination.

To confirm the accuracy of the categorisation of asymptomatic FSW, we randomly selected and manually reviewed the medical records of 200 consultations used in our study categorised as ‘asymptomatic’. Of those included, 95.5% were confirmed to have been correctly categorised as asymptomatic. Of the 200 consultations, nine (4.5%) were found to be incorrectly categorised as ‘asymptomatic’, as triage-notes indicated the client had disclosed symptoms. However, none of the 200 random cases reviewed involved diagnoses of herpes or warts.

## Discussion

Our study found that the additional genital examinations rarely identified anogenital herpes or warts in asymptomatic FSW. Indeed about 500 genital examinations were required to diagnose a single case. Previous studies have shown that examining asymptomatic females who were not sex workers was unnecessary and the findings from the present study suggest that this also applies to FSW [7–9]. Importantly, our study demonstrated that FSW accurately reported the presence of symptoms and did not conceal their herpes or warts. As a result of this data, MSHC has stopped examining FSW in 2019 unless they report symptoms. Asymptomatic FSW will now be encouraged to self-collect vaginal swabs for chlamydia and gonorrhoea screening. This change will significantly reduce consultation times for unnecessary examinations on FSW, and this additional time saving can be allocated to see more clients or to clients who are at a higher risk of HIV/STI. It is particularly important to improve clinic efficiency at MSHC as the service is currently operating at capacity without additional resources. The Sex Work Regulations also lists chancroid and donovanosis as STIs that would prohibit a FSW from working. However, these infections are not routinely screened for at MSHC given there have been only two cases of donovanosis since 2011 in Australia, and no female case of chancroid at our clinic in the past 40 years [10, 11].

There are several limitations that should be considered when assessing our study. First, our results are from a single urban sexual health clinic that may not be representative of all women working as sex workers in Melbourne. Moreover, the results of the study are specific to FSW within a legal and regulated licencing system where the prevalence of STIs is low (i.e. 3% for genital chlamydia and 1% for genital gonorrhoea) and the proportion of FSW who condom with their male client is relatively high (i.e. 97% for penile-vaginal sex) [4, 12]. As STI prevalence among FSW varies greatly, we highly recommend that other clinics complete their own investigation like that of the present study before implementing changes to clinical practice. Second, this was a retrospective study that utilised data not collected with the purposes of this study in mind. However, the data was collected using pre-set diagnosis categories previously shown to be accurate [6]. Finally, a review of 200 random consultations categorised as asymptomatic used in our study found that 4.5% (9/200) of consultations were misclassified as asymptomatic because the client had in fact self-reported symptoms to the clinician during clinical consultation. This suggests the true proportion of asymptomatic FSW diagnosed with lesions following examination may be marginally lower than our calculation of 0.21%.

The public health value of identifying visible warts or herpes lesions among sex workers who self-report asymptomatic is likely to be limited, this is because most human papillomavirus (HPV) and herpes infections are subclinical and transmission often occurs during subclinical infections rather than from visible lesions [13, 14]. There has been a substantial reduction and nearly elimination in genital warts and HPV genotypes 6/11 among young Australian women and men after the introduction of the national HPV vaccination program in 2007 [15–18]. However, there have been no studies examining the vaccination coverage and the prevalence of genital warts among female sex workers in Australia. Past studies have shown that the majority of female sex workers in Melbourne are born in Australia [12], and therefore it is reasonably hypothesised that most of them might have received the HPV vaccine from the national HPV vaccination program. Facilitating efficient screening is good for both sex workers and the general population and should not be complicated by unnecessary and expensive examinations. Improving the efficiency of STI services is becoming increasingly important as the rates of STIs in most industrialised countries are at their highest level in 30 years, and attracting additional funding for sexual health services is challenging [19–22]. The time saved by not examining FSW at our clinic can be allocated to individuals with a higher risk of acquiring HIV and STIs.
