# Correction: Are genital examinations necessary for STI screening for female sex workers? An audit of decriminalized and regulated sex workers in Melbourne, Australia

**DOI:** 10.1371/journal.pone.0232925

**Published:** 2020-05-04

**Authors:** The PLOS ONE Staff

The following information is missing from the Funding statement: EPFC is supported by an Australian National Health and Medical Research Council (NHMRC) Emerging Leadership Investigator Grant (GNT1172873). CKF and CSB are supported by an Australian NHMRC Leadership Investigator Grant (GNT1172900 and GNT1173361, respectively). The publisher apologizes for the error.
